# Managing unusual sensory experiences: A feasibility trial in an At Risk Mental States for psychosis group

**DOI:** 10.1111/papt.12323

**Published:** 2020-12-15

**Authors:** Guy Dodgson, Charlotte Aynsworth, Kaja J. Mitrenga, Chistopher Gibbs, Victoria Patton, Charles Fernyhough, Robert Dudley, Carina Ewels, Louise Leach, Ben Alderson‐Day, Stephanie Common

**Affiliations:** ^1^ Cumbria, Northumberland, Tyne and Wear NHS FT Greenacre Centre Ashington UK; ^2^ Cumbria, Northumberland, Tyne and Wear NHS Foundation Trust UK; ^3^ Psychology Deptartment Durham University UK; ^4^ Department of English Studies Durham University UK; ^5^ School of Psychology Newcastle University UK; ^6^ Tees, Esk and Wear Valley NHS FT Stockton‐on‐Tees UK

**Keywords:** at‐risk mental state for psychosis, hallucinations, psychological mechanisms, therapy

## Abstract

**Objectives:**

To conduct a feasibility study on a new, tablet‐delivered treatment for unusual sensory experiences in service‐users with an At Risk Mental States for psychosis.

**Design:**

A mixed method design was employed, using content analysis to investigate whether service‐users and therapists found the new treatment acceptable and helpful. We also collected data on the impact of treatment, but without a control group could not make any claims about effectiveness.

**Methods:**

Eligible participants were contacted before starting treatment and offered the chance to participate. Assessments were conducted before and after the treatment, which typically was completed in 4–6 sessions by an accredited CBT therapist. A structured interview was used to collect qualitative feedback.

**Results:**

Qualitative feedback suggested that the treatment was acceptable to service‐users and therapists, and the progression criteria were met for recruitment, retention, and adherence to treatment.

**Conclusions:**

The new treatment targeting subtypes of auditory and visual hallucinations was acceptable to service‐users and the benefits of addressing psychological mechanisms thought to contribute to hallucinations was supported by qualitative feedback.

**Practitioner points:**

- A novel treatment has been developed for unusual sensory experiences
- based on subtyping voices and using technology to help explain psychological mechanisms that may be linked to hallucinations.
- The treatment was acceptable to service users and therapists in At Risk Mental States for psychosis services
- with qualitative feedback supporting the approach.
- The treatment may be particularly useful in preventing the progressions of psychosis
- as people who have not developed fixed ideas about the origin of the experiences may be more open to alternative explanations

## Background

The concept of an At Risk Mental States (ARMS) for psychosis (Yung et al., 1996) was introduced to describe individuals at elevated risk of developing later psychotic disorders. ARMS has been operationalized in terms of three subgroups involving: (1) a brief episode of psychotic symptoms, lasting less than 7 days, that remits without treatment; (2) attenuated symptoms of psychosis; and (3) deterioration in functioning and a family history of psychosis (Yung et al., 2005). Several meta‐analyses (van der Gaag, Valmaggia, & Smit, 2014; Hutton & Taylor, 2014; Stafford, Jackson, Mayo‐Wilson, Morrison, & Kendall, 2013;) have suggested that interventions in ARMS can reduce the conversion to psychosis by 50%. These reviews, as well as National Institute for Health and Care Excellence (NICE) guidance (2014), recommended that people showing signs of an ARMS for psychosis should receive Cognitive Behavioural Therapy (CBT). However, a later umbrella review, noting the paucity of relevant evidence, suggested that no specific intervention has demonstrated superior efficacy (relative to any other) in preventing psychosis in ARMS individuals (Fusar‐Poli et al., 2019).

Unusual sensory experiences like hallucinations are relatively common in the non‐clinical population, meaning people can have these with no need for care (e.g., Johns et al., 2014; Toh, Thomas, Robertson, & Rossell, 2020). Nevertheless, hallucinations are a key element of the presentations of help‐seeking people attending specialist ARMS services (O’Connor et al., 2016) meaning that, for some, hallucinations are distressing and disabling. Auditory verbal hallucinations are also particularly associated with the risk of developing psychotic disorders in clinically high‐risk groups (Niles, Walsh, Woods, & Powers, 2019). However, what leads someone to make the transition to illness is unclear. It is not the presence of hallucinations alone, but is perhaps owing to precipitants such as increases in current stressors or the experience of recent trauma (de Leede‐Smith & Barkus, 2013; Larøi et al., 2012), changes to cognitive appraisals, or increases in other symptoms (e.g., anxiety, depression). It is doubtful that there is one single pathway to psychosis, which represents a broad category of experiences and symptoms, but there are likely to be dynamic interactions between hallucinatory experiences, delusional ideation, sub‐clinical negative symptoms, and affective changes that may predict subsequent transition to psychosis (van Os & Reininghaus, 2016).

Consequently, an intervention for people in the ARMS category that specifically and purposefully targets hallucinations may both help reduce the current frequency, distress and impact on functioning, but could also help prevent the transition to a first episode of psychosis. CBT for psychosis (CBTp) is helpful for dealing with voices to a modest degree (Turner, Burgess, Smit, Valmaggia, & van der Gaag, 2020) and has benefits for a range of other presenting issues like mood and anxiety. However, given the importance of hallucinations in ARMS, there is scope to focus on these important symptoms and to draw on models of what leads people to see or hear things others do not, in order to help to target causal mechanisms. Managing Unusual Sensory Symptoms (MUSE) is a novel treatment which aims to improve outcomes through (1) increasing treatment specificity by supporting understanding that hallucinations may arise from different cognitive and perceptual processes); (2) drawing on current psychological models of how the brain works, highlighting putative mechanisms which may be implicated in the development of hallucinations; and (3) using technology to demonstrate key concepts, make the treatment accessible and increase access to the intervention. For example, with regard to subtypes of hallucinations, the concept of Hypervigilance Hallucinations (Dodgson & Gordon, 2009), helps explain why expectancy or prediction can lead people to hear things in ambiguous background noise, particularly when someone feels threatened (Dudley et al., 2014). Along with other theoretical perspectives, MUSE draws on the Predictive Processing Framework to explain how prior expectations can influence perception and uses interactive tasks to highlight the importance of prediction to sensory processing (Alderson‐Day et al., 2017) but also how predictions can lead to errors (Hohwy, Roepstorff, & Friston, 2008). The treatment was initially developed by clinicians in Early Intervention in Psychosis (EIP) service in the North East of England, but has been extended with input from the Hearing the Voice project, and the content reflects the clinical and theoretical background to the manual. The treatment can be accessed via the following link (https://web.ntw.nhs.uk/gsh/VH ).

Initially the treatment was developed for people experiencing a first episode of psychosis and was used as a brief intervention (4–6 sessions focussed on hallucinations) within a larger package of psychological therapy as part of an initial feasibility study (Dodgson et al., 2020). This showed the intervention to be acceptable, with high scores for on the Satisfaction with Therapy and Therapist Scale (Oei & Shuttlewood, 1999). The feasibility study included participants from secondary care services who had longstanding psychosis, and it was noted that clinicians from EIP services found the intervention particularly helpful, consistent with higher rates of first‐episode participants completing the intervention. In addition, initial findings suggested that MUSE appeared to be more effective for people who had not developed fixed or delusional ideas about their experiences, which may become a secondary treatment issue and, owing to processes like rumination (Lebert, Turkington, Freeston, & Dudley, 2020), may make people less open to alternative explanations. Finally, MUSE highlights processes that may increase unusual sensory experiences, such as rumination, thought suppression, and isolation after starting to experience voices, which may be addressed in therapy. MUSE may thus be particularly suited to arresting the progression of hallucinations, supported by the fact that feedback from clinicians who used MUSE suggested it was particularly helpful for people in the early stages of their experiences. These features of the package make MUSE potentially highly relevant for therapeutic intervention in the ARMS population.

At Risk Mental States services are recommended to provide 10–16 sessions of CBT. In the UK, however, not all areas have ARMS services, not all services can provide CBT and not all people will accept or want CBT. A range of effective and accessible treatments are therefore needed. The aim of the present work was to conduct a feasibility study, with key outcomes being acceptability (assessed by service‐user and therapist interviews and rating scales) along with recruitment, retention, and adherence to treatment. In addition, outcome data were collected to identify which measures may be appropriate to use in a definitive trial and also to identify if there was a ‘signal of efficacy’ that may suggest that the treatment may be effective.

## Method

### Participants

Twenty‐two participants were recruited from EIP teams in Tees, Esk & Wear Valley NHS FT and Cumbria, Northumberland Tyne and Wear NHS FT. The average age was *M*(*SD*) = 24.18 years (*SD* = 4.52), and 19 were male. 19 participants completed therapeutic intervention and pre‐ and post‐assessment measures, two participants transitioned to first‐episode psychosis prior to commencing therapeutic intervention and one transitioned after starting therapy but prior to post‐therapy assessment. While the former two were excluded, the latter participant was nevertheless included in our analysis on an intention‐to‐treat basis. Potential participants were identified from a cohort of service‐users commencing an ARMS pathway following a screening assessment using the Comprehensive Assessment of At Risks Mental State (Yung et al., 2005). Services users on the ARMS pathway who were appropriate for cognitive behavioural therapy were offered the opportunity to take part in the trial. Inclusion criteria for participation were meeting criteria for ARMS, with auditory verbal hallucinations presenting as an attenuated positive symptom; having identified hallucinations as target problem for psychological therapy; and being aged 16 years or over. Exclusion criteria were organic basis for hallucinations; intellectual disability; insufficient command of English language and primary diagnosis of substance misuse. Participants received a gift voucher for completing the assessments. Ethical approval for this study was given by NRES Committee Leeds East (REC Reference Number: 18/YH/0433). The authors have abided by the Ethical Principles of Psychologists and Code of Conduct as set out by the APA.

### Treatment manual

The therapists were provided with a treatment manual in a computer tablet format. The manual contained eight modules. The first two modules (What are Voices and How the Mind Works) were designed to inform about and normalize the experience of unusual sensory experiences. The third module (Assessment) was designed to help identify the relevant subtype of voices (the key criteria used are outlined in Dodgson et al., 2020). There were also five treatment modules relating to different subtypes: Inner Speech ‐ AVH (IS‐AVH), Memory Based‐AVH (MB‐AVH), Hypervigilance ‐ AVH (HV‐ AVH), Visual Hallucinations and Sleep. (See supporting informations for a list of the topics within each module). Following service‐user feedback in the previous study, MUSE was redesigned to enable the intervention to be service‐user facing and made more flexible for therapists to either linearly follow the treatment or to change the order based on their judgement. The redesign was led by an expert‐by‐experience who is part of the research team (CG).

Trial therapists received a 2‐day training course and monthly group supervision. Each treatment section contains psychoeducational materials in multi‐media format to illustrate concepts and intervention approaches. The manual was designed to be used in a bespoke format driven by identification of relevant subtypes and individual service‐user’s priorities and preferences.

### Procedure

Potential participants were identified at acceptance into the ARMS pathway or on referral to the psychological therapists, and were then approached by a Research Assistant (RA) to discuss whether they were willing to participate. If interested in participating, the RA forwarded a Participant Information Sheet and arranged to meet the individual to further discuss participation, complete the consent process and undertake the baseline assessment. Psychological therapists were all accredited CBT practitioners with at least 2 years’ experience and were already working within the ARMS pathway. Therapists were asked to provide an assessment and formulation to the participant and then to use the manual in the first two to four therapy sessions, prior to therapeutic focus on other target problems and goals unrelated to hallucinations. This design ensured that service‐users had adequate exposure to the treatment manual while also allowing for therapy to address the primary goals and needs of service‐user. On completing MUSE, the therapist informed the RA, who then arranged to complete the post‐treatment assessment. This was a small feasibility study and it was not possible to include a control group.

### Outcomes

As this is a feasibility study, our primary outcomes were referral, recruitment and retention rates, acceptability of treatments (determined through assessing discontinuation rates, service‐user satisfaction, and qualitative analysis with participants’ and therapists’ views on the treatment) and adherence to the MUSE intervention. To determine feasibility, we applied 3‐stage progression criteria (Avery et al., 2017) relating to recruitment, retention to post‐treatment assessment and adherence to MUSE. The progression criteria were devised by the research group and are as follows:

1. Recruitment ≥80% of eligible participants consented into the study (green), recruitment within 79–60% of consent (amber), recruitment <60% of consented (red).
2. Retention of participants within the study with baseline and outcome assessments at primary end point (end of treatment) ≥80% of primary outcome completed (green), 79–60% of primary outcome completed (amber), <60% of primary outcome completed (red).
3. Satisfactory delivery of adherent therapy to ≥80% of participants receiving MUSE (green), 79–60% of participants receiving MUSE (amber), <60% of participants receiving MUSE (red). Satisfactory delivery of adherent therapy is operationalized as completing at least one of the four unusual sensory experiences modules. Therapists completed an adherence checklist after each session in order to track which components of the manual were utilized.

#### Scales

1. *Satisfaction with Therapy and Therapist Scale* (STTS; Oei & Shuttlewood, 1999 **)**, the 11‐item scale was used to assess overall acceptability of the therapeutic interaction. The measure includes questions relating to satisfaction with therapy and therapist (e.g., ‘I am satisfied with the quality of the therapy I received’). The scale was found to have strong validity and reliability (mean Cronbach’s alpha = .90; Oei & Shuttlewood, 1999).
2. *Psychotic Symptom Rating Scales: Auditory Hallucinations Subscale* (PSYRATS; Haddock, McCarron, Tarrier, & Faragher, 1999 **)** is a clinician administered semi‐structured interview of hallucinations. The PSYRATS has been used extensively as an outcome measure in CBTp research in auditory hallucinations. The scale includes detailed ratings of hallucinations (e.g., relating to frequency, duration or negative content of voices), rated on a scale from 0 (‘No problem’) to 4 (‘Maximum severity’). The scale shows good reliability and validity (Drake, Haddock, Tarrier, Bentall, & Lewis, 2007).
3. *The Comprehensive Assessment of At Risk Mental States* (CAARMS; Yung et al., 2005 **)** is a clinician administered 27‐item semi‐structured interview that assesses attenuated psychotic and psychotic symptoms. The CAARMS was designed to identify the attenuated positive symptom thresholds necessary to fulfil ARMs criteria. The perceptual abnormalities section was administered to assess hallucinatory like experience in six sensory modalities. Examples of questions include: ‘Do you ever hear things that may not really be there?’ or ‘Do you ever get strange feelings on, or just beneath, your skin?’.
4. *The Depression, Anxiety and Stress Scales* (DASS‐21; Lovibond & Lovibond, 1995 **)** is a 21 item self‐report scale and was used to assess changes in symptoms of emotional distress, stress, anxiety and depression. It consists of 21 items (e.g., ‘I couldn’t seem to experience any positive feeling at all’) that are rated on a Likert scale ranging from 0 (‘Did not apply to me at all’) to 3 (‘Applied to me very much or most of the time’). The scale shows excellent reliability and validity (Coker, Coker, & Sanni, 2018).
5. The short‐form of the *Choice of Outcome In CBT for psychoses* (CHOICE; Greenwood et al., 2010 **),** is a 12‐item service‐user developed scale and was used to assess progress post‐intervention in achieving therapy‐related goals. Examples of items include statements relating to ‘Ways of dealing with distressing experiences (e.g., beliefs, thoughts, voices)’ or ‘Positive ways of thinking’.
6. *The Investigating Choice Experiments Capability Measure for Adults* (ICECAP‐A; Flynn et al., 2015) was used to measure changes in recovery related functioning. It comprises of five questions relating to five dimensions of wellbeing: attachment stability, autonomy, achievement and enjoyment. Each statement is rated on a scale from 0 (e.g., ‘I am unable to feel settled and secure in any areas of my life’) to 4 (e.g., I am able to feel settled and secure in all areas of my life’).
7. *The process of recovery* (QPR Neil et al., 2009), is a 15‐item service‐user developed scale used to assess recovery from psychosis outcomes framed in terms of the CHIME principles. The items (e.g., ‘I feel that my life has a purpose’) are rated on a 5‐item scale, ranging from ‘Disagree strongly’ to ‘Agree strongly’. The questionnaire was found to have strong validity and reliability (Law, Neil, Dunn, & Morrison, 2014).

All data from these scales that were used in statistical analyses are available to download here: https://osf.io/h9rsp/?view_only=41e82d95b80e4be08f1242faa5b729f3.

### Qualitative analysis

Each of the participating service‐users and therapists completed a short exit interview with a research assistant on completion of the study. To ensure consistency, a structured interview was used, consisting of eight questions about the experience of therapy (nine for therapists) and four questions on the experience of taking part in the research. The questions were selected based on clinician and service‐user feedback from a study using a previous iteration of MUSE (Dodgson et al., 2020), focusing on the overall experience of therapy, acceptability of the tablet, pros and cons of the intervention, and their experience of research. Although service‐users and therapists were asked slightly different questions in their interviews, their responses were analysed together to explore points of overlap and divergence in their experience of using the intervention.

Interview responses were then analysed by two researchers and a basic coding frame was devised using a conventional content analysis in attempt to capture the main issues brought up by participants (i.e., codes were not chosen in advance or dictated by theory, as in directed content analysis; Hsieh & Shannon, 2005). However, because of the structured nature of the interviews, and because we intended to report quantitative summaries of the codes derived, our epistemological position was closer to a positivist understanding more typically associated with quantitative content analysis (Krippendorff, 2018).

Two researchers (CA and VP) independently developed descriptive codes for the dataset. Codes were then discussed and refined, and a third researcher (BAD) was consulted on code development. Both researchers then applied the agreed codes to 20% of the dataset for parallel coding to ensure reliability, then the remaining data was coded. Inter‐rater agreement and reliability were acceptable to good (agreement = 91.62%, kappa = 0.71). The subsequent codes were grouped into three main topics: using MUSE, impact on clients, and impact on therapists. Each response could only receive each code once, but each answer could have different meanings so it could fit across multiple categories. Development, analysis and reporting of codes was conducted bearing in mind the positions of the two coders – one a clinical psychologist, the other a public engagement and involvement expert. This may have influenced their analysis by foregrounding issues of service‐user understanding, positive feedback, and accessibility. Both reviewers were particularly interested in whether the intervention provided clients with new ways of understanding their experiences, had a normalizing effect, and reduced self‐stigma and feelings of isolation.

## Results

**Figure 1:**
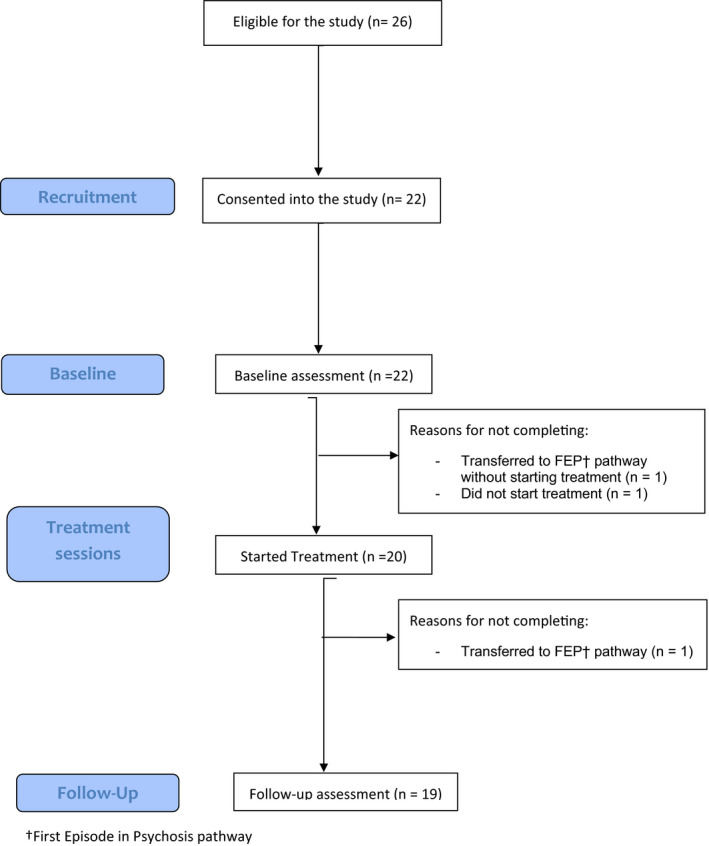
Completion flow diagram for participants recruited, receiving treatment, and completing MUSE. ^†^First Episode in Psychosis pathway.

Twenty‐six participants were identified as eligible with 22 consenting (84% recruitment, green zone) and 19 participants completing therapeutic intervention and pre and post‐assessments (86% retention, green zone). The high recruitment rate ensured that the participants reflected the referrals to the service and resulted in a high proportion of male participants (86%), as would be expected in a service aiming to reduce the onset of psychosis. Recruitment and retention were high through the trial, possibly reflecting rapid access to an intervention that directly targeted the participant’s reason for help seeking. In the exit interviews, 79% of participants suggested that they would have agreed to participate even if they may have been randomized to treatment as usual condition. One participant transitioned to first‐episode psychosis prior to commencing therapeutic intervention and one transitioned after commencing therapy, but prior to post‐therapy assessment: baseline data for the latter was therefore taken forward and included in the final analysis on an intention to treat basis. Figure 1 shows the CONSORT diagram for the study. Referral rate was above prior expectations enabling the study to close for recruitment earlier than expected. The participant who did not complete the treatment was transferred to the FEP pathway, after their mental health deteriorated following a relationship breakdown; this was recorded as an Adverse Event, in accordance with the Standard Operating Procedure from the Sponsoring Trust, and based on NIHR Guidance (this was the only reported Adverse Event in the study).

Adherence checklists were completed in order to help assess feasibility of delivery of MUSE. Checks were completed for 17 service‐users in the study (see Table 1). On average therapists used 3.94/8 modules during treatment, with one AVH subtype being the mode number used in 8 cases (7 used two subtypes, one used all three subtypes, and one only focused on the visions module). The progression criteria were met, with 17 (85% adherence, green zone) of service‐users completing at least one unusual sensory experience module. As Table 1 indicates, the introductory modules on ‘What are voices?’ and ‘How the mind works’ were used in almost all cases, typically by session 2–3 with the service‐user. Inner speech was the AVH subtype used most, followed by hypervigilance and memory subtypes. When a module was selected, the vast majority of topics within that module were reported as being used, with the exception of the hypervigilance and visions modules, where some modules repeated content from earlier modules.

**Table 1:** Adherence to therapy across MUSE modules

Module	Used (*N* = 17)	Mean	
		Topics (%)	First session used
Introductory			
What are voices?	14	98.57	2.43
How the mind works	17	98.82	2.94
Assessment	9	100.00	4.56
AVH^a^ subtypes			
Inner speech	15	85.13	5.13
Memory	2	100.00	7.50
Hypervigilance	8	50.00	5.75
Additional			
Visions	4	61.54	4.00
Sleep	2	100.00	3.00

Note

^a^ Auditory verbal hallucinations.

### Qualitative analyses

Table 2 displays the codes used to classify responses from both clients and therapists.

**Table 2:** Using MUSE

Description	Service‐users *n* = 19 Therapists *n* = 7
	Example
MUSE was simple and straightforward to use	Service‐users (10 Positive, 0 Negative comments) ‘Piece of cake!’ (P16) ‘The actual tablet using was straightforward … and it was good to be able to look at the videos and stuff as well, from the actual like research’ [P 9]. ‘It made it easier than like just having like loads of papers and loads of forms and stuff like that …’ (P 15)
	Therapists (5 Positive, 1 Negative comment) ‘It was really easy to be able to dip in and out of modules, being able to kind of find what I needed to find, so it was actually quite easy to use’ (T 1) ‘…there were a few kind of like just … just minor issues, things like sound … things like the videos.’ (T 7)
MUSE was well‐structured and well‐organized, with a clear pathway through different modules on assessment, psychoeducation, formulation and intervention. Materials were easy to find	Service‐users (1 Positive comment) ‘… it was good, it was structured, it seemed to be going somewhere’ (P 9) Therapists (5 Positive and 1 Negative comment) ‘I liked the way that it provided a gradual build of the service‐users’ knowledge and … allowed them to kind of develop from standing. What was specifically helpful for me was that it reassured me that I hadn’t missed anything’ (T 7) ‘I think sometimes things come up and you’re thinking, oh that’s in that other bit, and … it kind of loses something if you’re there going, right, I just need to find …’ (T5)
	Therapists (5 Positive and 1 Negative comment) ‘I liked the way that it provided a gradual build of the service‐users’ knowledge and … allowed them to kind of develop from standing. What was specifically helpful for me was that it reassured me that I hadn’t missed anything’ (T 7) *‘I think sometimes things come up and you’re thinking, oh that’s in that other bit, and … it kind of loses something if you’re there going, right, I just need to find …’* (T5)
MUSE content was useful and relevant to the clients and therapists’ needs	Service‐users (19 Positive, 1 Negative comment) ‘It helped me understand different stuff. …Yeah, where voices were coming from and what they meant’ (P 6) ‘Yeah, it was really good, ehm, really helpful, ehm … you got to see like different reasons for why I was like hearing voices and stuff. So it was really helpful for me’. (P 7)
	Therapists (7 Positive comments) ‘it was really nice having an iPad that you just carry, you just take it out and you just open it up and use. And kind of having lots of clips and different things that you know we were able to use’. (T 3) ‘I just found it a really, really useful tool, sort of really relevant to the client group, you know an easy process to follow with the slides and the assessment modules’ (T 2)
The information on MUSE was accessible and easy to understan	Service‐users (7 Positive, 5 Negative comments) ‘I like watching videos, I think it was easier to understand than when she [the therapist] was talking’. ‘Erm … because you kinda have images, videos, you kinda …. Like learn a little bit more than if you just did it like through talking or … you took in a bit more’. (P 15) ‘I think some of the like kind of metaphorical language just comes across a bit patronising … I think that there’s some ways you can try and make it kind of more … personal or easier to understand’ (P 19) ‘the information in it is very good, the delivery of it’s pretty poor ….’ (P 12)
	Therapists (3 Positive, 2 Negative comments) ‘Some of the things that I found harder to explain verbally or without a … visual prompt, they came across so much better being well prepared on the slide. Even things that were actually quite simple you could see the patients kind of really getting it from … it being in written down form or picture form’. (T 6) ‘I think it was pitched at the right level to sort of get a large group of people really’ (T 6) ‘I mean my … guy, I don’t think he’s probably got the highest intellectual sort of ability as … so I think some of the things were a little bit … academic for him’ (T 3)
The information and multi‐media resources embedded in MUSE were interesting and engaging	Service‐users (10 Positive, 6 Negative comments) ‘It was good, it was interesting … because you kinda have images, videos’ (P 15) ‘The videos on the tablet … they were a bit long and … well they were just very boring’ (P 10)
	Therapists (3 Positive comments) ‘It was nice having the different clips that kind of talked about things and … I think those were the things that people like’ (T 4) ‘the things that my patient really loved with the videos were the things that he could participate in doing’ T3.
MUSE requires training and preparation before use with clients	Service‐users (1 Positive comment) ‘I wouldn’t recommend diving straight in on a tablet, you know with someone that you hadn’t spoken to, there was a good few sessions that we had before we even … you know looked at the … the programme, which I think is definitely needed, otherwise you’re just sat in a room with a person you don’t know, looking at a computer screen’.(P 9)
	Therapists (4 Positive comments) ‘I think it takes a bit of time to get used to, and you certainly need to have done some prep … you couldn’t go into the session having not kind of got your way round it’. ‘… as long as you sort of flick through it before the session and think about what vague area you were going to be going on, it was easy enough to find the bits that were relevant to what you were talking about’. (T 3) ‘I think if I’d started off with the tablet, I think it might have impeded my ability to build a relationship with him. But because I’d already had a relationship … I think it was sort of an OK took to be using, but I don’t think I’d have liked to have used it from day one because then I think it might have hindered it a little bit’. (T3)
Therapists and clients would recommend MUSE as an intervention for people with psychosis	Service‐users (18 Positive, 1 Negative comment) In answer to the question, would you recommend MUSE to others: ‘Completely. Because it showed me that … it wasn’t just me thinking of these things, it was showing us … that it was … my mind playing tricks on us’. (P 2) ‘No….Purely on the delivery of it’. (P 12)
	Therapists (7 Positive comments) ‘Yeah, absolutely, yeah. It’s definitely something that I think could be … be used quite a bit, especially in EIP definitely’. (T 1)

#### Using MUSE

Five of the therapists and 10 of the service‐users made positive comments about how the treatment was easy to use, with only one negative comment from a therapist. Apart from a few technical issues (relating to volume, device battery life, and Wi‐Fi access), the delivery of the therapy was simple and straightforward. Therapists found the manual well‐structured and well‐organized, specifying that there was a clear pathway through the modules, and that psychoeducation materials were mostly easy to find. For therapists, the modular format of MUSE improved the structure of sessions and helped to keep focus. Three therapists reported difficulties navigating the manual, emphasizing that finding information quickly and efficiently required a degree of training and preparation.

All therapists and service‐users found the informational content of MUSE to be highly useful and relevant to their needs. For clients, ‘talking about my problems in my past life and how it’s affecting us in the present day. It showed us that…your mind can play tricks on ya and sometimes you think other people are talking about ya, but it’s not, it’s just your mind putting these thoughts into your head’ (P2). The intervention provided new ways to understand unusual experiences, facilitating insight into the way in which hallucinations can be linked to previous life events (e.g., trauma), and cognitive processes such as inner speech, memory and the role of prediction in perceptual processing. Therapists reported that MUSE was a useful and effective clinical tool. Consolidating psychoeducation materials and resources in one device provided a toolkit ‘that you can just dip in and dip out of when you want any examples … a really good clinical tool.’ (T3)

The majority of comments were positive about the material being accessible, easy to understand, and presented suitably for the target audience. For therapists, one of the advantages was that the multi‐media resources (e.g., videos and talking points) ‘allowed me to do is to deliver some quite complex concepts in a really kind of accessible way’ … (T7).

Clients also commented that MUSE made psychoeducation materials easier to grasp ‘because you kinda have images, videos, you kinda …. like learn a little bit more than if you just did it like through talking’. (P15)

However, five service‐users suggested that MUSE content was inaccessible because it was presented in an alienating language or style: ‘If you’re not very good at classroom learning … I don’t think it is going to appeal’ (P12). Two of the therapists raised a similar concern, suggesting that some content was too ‘academic’ for their clients.

Finally, most of the therapist and service‐users comments reported that MUSE was interesting and engaging, with clients noting that the videos and interactive activities were particularly enjoyable. However, six service‐users described the resources as ‘boring’ or ‘repetitive’. Nevertheless, 94.7% of clients and 100% of therapists stated they would recommend MUSE to others, which tallied with the service‐users overall satisfaction scores on the STTS which were very good, with a mean score of 49.47 (*SD* = 5.60) out of a maximum of 55 (mean item response = 4.50/5, indicating strong agreement).

#### Impact of MUSE

Tables 3 and 4 show that MUSE had several impacts that were shared by both service‐users and therapists. High numbers of service‐users and therapists reported that the tablet increased engagement with therapy, making it easier to form a strong therapeutic relationship, improving interest and attendance, and helping clients to feel less anxious. For some clients, MUSE prompted engagement with therapy outside sessions, inspiring them to research topics and additional sources of support.

**Table 3:** Impact on clients

Description	Service‐users *n* = 19
	Example
Personal stories and other resources embedded in MUSE reduced self‐stigma and feelings of isolation	8 Positive, 0 negative comments ‘Yeah, finding out you’re not the only person in the world hearing voices, and that you're not actually going insane’ (P 1) ‘… makes them [the voices] seem you know far more explainable than they were. Beforehand it was like this weird, whacky kind of green thing that makes you a complete nutcase and now it's like … feedback in the brain or something that can be explained, which is good’. (P 9)
Use of MUSE reduced anxiety and improved ways of coping and living with unusual experiences. Voices sometimes disappeared altogether	8 Positive, 0 negative comments ‘It helped me to remain calm when I experience … oh I can't think of the word … the way that I feel I guess …’(P13) ‘… it made sense like why the voices were happening, now they're not there anymore. Yeah, it made a lot of difference’. (P 17)
MUSE was more effective and engaging than other talking therapies (e.g., standard CBTp)	11 Positive, 2 negative comments ‘I just think it is a lot more helpful than just sitting talking to someone’ (P 18) ‘Personally, I think that … using the tablet is more beneficial than not using the tablet. … [People who don’t have the tablet] can't see … like they can't visualise and hear what's going on … like what the tablet's about, like what's entailed with the therapy’ (P 4)
MUSE facilitated a strong working relationship with the therapist, reduced anxiety and increased engagement in sessions	18 positive, 2 negative comments ‘I didn't feel like I was sat in a prison cell. Being interrogated by … I felt like it was more … that I was not going to like a meeting, but I was more like … didn't feel like I was on edge as much’. (P 3) ‘Rather than sitting there and talking with the therapist, I was spending the majority of time reading something, then asking questions and getting answers’. (P 12)
MUSE made it easier for the client to communicate their feelings and experiences and provided starting points for exploring issues in more depth.	6 Positive, 1 negative comment ‘… it helped him [my therapist] I think almost like relate a bit more to what I was trying to say, because sometimes I found it hard to kind of work out what I wanted to … to say or I found it hard to explain something. So if we were going through the table, it was easy for me to just point …’ (P 7) ‘I'd be very confused about what we were talking about’ (P 14)
MUSE legitimized psychoeducation materials and improved trust in psychological and neuroscientific explanations of unusual experiences	2 Positive, 0 negative comments ‘If I hadn't seen like the stuff on the tablet about how ya mind can make different things, I wouldn't have been able to understand it better in a sense because … yeah, I'm getting told these things off a person but … how do I … where's like the proof that I can look at to say, right, I can understand that, I can go through it and then … it all links up together, which you don't get that if you're just talking to someone, you don't physically get to look at it and see … the ways in which it works’ (P 2) ‘It showed me that she wasn't just telling us all this, it was proof on, not paper but obviously a tablet’. (P2)

**Table 4:** Impact on therapists

Description	Therapists *n* = 7
	Example
Use of MUSE enhanced therapists’ knowledge, skills, and confidence in working with people who have unusual sensory experiences	5 Positive, 0 negative comments ‘… for me as part of my kind of learning and kind of understanding of things … getting access to the resources … was really, really helpful. And actually, from my perspective, that's helped you know kind of my development as a therapist’. (T 4) ‘What was specifically helpful for me was that it reassured me that I hadn't missed anything. … And I like reassurance that I've … been thorough and as robust as I can be, and so it … provides me with the reassurance that the service user had gained what he needed to, and obviously that it was evidence based’. (T 7)
The psychoeducation materials and structure of MUSE improved the way the therapist developed a formulation for the client.	7 Positive, 0 negative comments ‘I was able to look at the theory, talk to the service user about kind of how that fit with their experiences, make some notes that we then like added to the formulation, rather than doing the formulation just in one session, it was kind of growing every week. So I do think it helped with that’. (T 1) ‘just working your way through those modules and then allowing the time to reflect on the … on the service‐users' experiences. So yeah, it did definitely help with the formulation’ (T 2)
MUSE can be adapted to individual needs and allows therapy to be more client led	5 Positive, 0 negative comments ‘What I did was I allowed the service user to drive the tablet and therefore to drive the pace as well. … And I think that gave them some ownership over the process’. (T 7) ‘I quickly learned that I needed to go on a different pace for … people in terms of their understanding. So for some people we would kind of go through a module each session, for other people it was really breaking down them modules. But again, that was quite easy to … to work out, depending on who I was talking to’. (T 1)
MUSE increased engagement in sessions, improved the therapeutic relationship and helped clients to feel more comfortable	6 Positive, 0 negative comments ‘… it did help with the relationships, got really positive feedback from it. Attendance rate was quite good, so that's always a good sign’ (T 2) ‘… you were kind of directing your attention to the device, so I think people felt more comfortable with what we were talking [about] because it wasn't like having to have that kind of eye contact and things’. (T 1)
MUSE improved communication between therapist and client, making it easier to explain psychological and neuroscientific theories in accessible ways	4 Positive, 0 negative comments ‘I think what it allowed me to do is to deliver some quite complex concepts in a really kind of accessible way. And it's … so most of the stuff was … the content was content that I was aware of but I don't think that I was particularly succinct or articulate in explaining them to patients’ (T7) ‘It was really handy to have it as something that could start discussions on things’ (T 4) ‘I think what was useful was being able to use the tablet to start off a conversation using the theory but then being able to kind of adapt that to people's personal experiences. So kind of using that as a starting point but then being able to have a conversation following out of that’ (T 1)
MUSE legitimized therapists' explanations of why unusual experiences were occurring and made them more powerful	4 Positive, 0 negative comments ‘I think feedback from clients is [they] kind of like … the kind of … legitimacy of something that's on a computer. So … [one of the clients] said 'oh I'm not being funny but like … it's more believable him saying it on the video, than … just you and I talking about that …’ There's something about being a product, it being something that's like … like produced in that way that people perhaps find the information a bit more compelling than they would if it's just in a dialogue’. (T 5)

Several service‐users and therapists reported that MUSE improved communication, making it easier for clients to share their feelings and experiences, and providing starting points for discussion. Therapists commented that the tablet‐based resources lent legitimacy and credibility to psychological and neuroscientific explanations of unusual experiences, ‘So it was some random person saying it, not just my therapist who I was sat with who kind of has to say that’ (P2).

##### Impact on service‐users

A number of service‐users described the manual as having a specific impact on them (see Table 3). Normalization was a key theme to emerge, with eight service‐users volunteering that MUSE contributed to a reduction in feelings of isolation, for example P7 suggested that ‘other people talking about their own experience just made you feel like you weren't by yourself’ and self‐stigma, ‘Beforehand it was like this weird, whacky kind of green thing that makes you a complete nutcase and now it's like … feedback in the brain or something.’ (P9)

Service‐users also reported that the intervention enhanced daily functioning and improved ways of coping with unusual experiences. Significantly, one participant no longer heard voices after the treatment: ‘… it made sense like why the voices were happening, now they're not there anymore … it made a lot of difference.’ (P17)

Ten service‐users made positive comments that ‘Using the tablet is more beneficial than not using the tablet. … [People who don’t have it] … can't visualise and hear what's going on … like what's entailed with the therapy.’ (P4)

Only two service‐users suggested that they preferred other forms of talking therapy to MUSE, citing difficulties concentrating and a preference for less structured forms of therapy as reasons.

##### Impact on therapists

For therapists, MUSE offered a number of benefits (see Table 4). Five reported that it increased their knowledge, skills, and confidence, for example T6 stated ‘I can see that [it’s] affected all of my practice … It's improved my knowledge, it's improved my confidence and … I think the patients get a lot out of the way it’s represented’.

All therapists agreed that MUSE improved formulation development. Firstly, the manual’s modular structure provided a ‘step‐by‐step’ approach to understanding the factors underlying the clients’ experiences. Secondly, the explanations of different AVH subtypes in MUSE allowed for greater specificity in the formulation.

Adaptability was another significant theme. Five therapists said that MUSE can be adapted to individual needs, enabling therapy to become more client led. It is possible to progress through the modules at different speeds, allowing the service‐user to ‘set the pace’ and to pick and choose the materials they would like to explore in more depth. No therapist reported any negative impacts. One concluded that, in their view, clients responded better to MUSE than to standard CBT ‘Users responded well to the iPad … possibly more so than … your CBT that you normally do’ (T2).

It was also noteworthy that participants spontaneously made comments that supported the underlying rationale for MUSE. Firstly, feedback suggested that the subtypes were meaningful to participants, ‘so I remind myself that I could just be being hypervigilant and just listening out for noises in/and my brain creating noises…which has helped me remain calm’ (P13). Secondly, MUSE draws on current psychological models of how the brain works and attempts to explain often complex theories that may explain the mechanisms implicated in the development of hallucinations. For example, P15 suggested ‘Yeah, yeah, it definitely helped me understand a lot of like … ehm … where it was coming from and it was more internal for me than it was like … maybe external’.

Therapists and participants clearly valued access to material that made these theories more accessible, ‘If I hadn't seen like the stuff on the tablet about how ya mind can make different things, I wouldn't have been able to understand it better in a sense because … yeah, I'm getting told these things off a person but … how do I … where's like the proof that I can look at to say, right, I can understand that, I can go through it and then’ (P2). Thirdly, the use of technology is supported in the quote above and there were several references to how seeing things helped understanding. Interestingly, it also seemed to give additional weight to the content, that it had been put on a tablet, ‘It showed me that she wasn't just telling us all this, it was proof on, not paper but obviously a tablet’ (P2). One therapist said ‘[one of the clients] said 'oh I'm not being funny but like … it's more believable him saying it on the video, than … just you and I talking about that’. The modular structure also promoted a consistent and thorough intervention ‘just working your way through those modules and then allowing the time to reflect on the … on the service‐users' experiences. So yeah, it did definitely help with the formulation’ (T2).

### Changes in symptoms

To explore the appropriateness of measures for further trials, baseline and follow‐up scores of auditory hallucination total, distress total and delusions total from the PSYRATS, were identified as likely primary outcome measures (see Table 5). PSYRATS scores showed similar reductions, with the largest change being observed for auditory hallucinations total score (*d* = 0.77). Despite a medium effect size (*d* = 0.42), delusion scores on the PSYRATS did not significantly reduce from baseline, although it should be noted that only 5/22 participants scored above zero at baseline on this measure, and two at follow‐up (suggesting insufficient incidence of delusional ideation in this ARMS cohort). Comparisons were made on the other outcome measures including the auditory and visual items of the CAARMS. Each of the CAARMS outcomes showed significant reductions from baseline, with the largest effect sizes being observed for auditory and visual severity ratings (*d* = 0.70–0.77), and, notably, improvements in current functioning scores (*d* = 1.55). There were no clear differences in improvement for auditory compared to visual experiences. Improvements in DASS scores were also significant, with the largest reductions being observed for depression scores (*d* = 0.87).

**Table 5:** Outcome measures

	Baseline		Follow‐up		*p*	*d*
	*M*	*SD*	*M*	*SD*		
CAARMS^a^						
Auditory severity	4.60	1.23	3.15	2.18	.003	0.77
Auditory frequency	3.60	1.47	2.55	1.88	.023	0.55
Auditory distress	67.50	27.70	44.25	40.14	.016	0.59
Visual severity	3.20	1.64	1.75	1.89	.005	0.70
Visual frequency	2.90	1.52	1.90	2.15	.025	0.54
Visual distress	50.85	36.62	21.00	34.93	.008	0.66
SOFA^b^ (Current)	53.60	9.13	68.00	14.46	<.001	‒1.55
PSYRATS^c^						
Auditory Hallucinations	26.30	9.16	17.25	13.33	.003	0.77
Distress	13.90	5.56	9.45	7.19	.010	0.64
Delusions	3.25	5.95	1.55	4.78	.077	0.42
DASS^d^						
Depression	12.75	5.35	8.20	5.16	< .001	0.87
Anxiety	11.25	4.98	8.50	5.10	.020	0.57
Stress	14.05	4.61	10.35	5.15	.012	0.62
ICECAP‐A (tariff)^e^	0.51	0.19	0.66	0.19	.004	‒0.73
CHOICE^f^						
Mean severity	3.99	1.36	5.86	1.91	< .001	‒0.88
Mean satisfaction	3.39	1.68	5.98	2.45	< .001	‒0.94

Notes

^a^ Comprehensive Assessment of At Risk Mental States.

^b^ Social and Occupational Functioning Assessment Scale.

^c^ Psychotic Symptom RATing Scale.

^d^ Depression, Anxiety and Stress Scale.

^e^ ICEpop CAPability measure for Adults.

^f^ CHoice of Outcome In Cbt for psychosEs.

Post‐treatment mean score on the QPR was 40.63 (10.94), representing 67.71% of maximum score, or a mean response of 2.71/4. While this may seem a relatively modest level of recovery, scores for capability nevertheless significantly improved on the ICECAP‐A tariff (*d* = −0.73). Moreover, scores on the CHOICE (in which participants rate their goals for therapy and recovery, and can specify their own aims from treatment) showed some of the largest shifts from baseline, with effect sizes above 0.9. The measures used appeared appropriate except for the delusions scale of the PSYRATS, which was only attempted by 16/19 people at follow‐up, and with many participants scoring zero. Aside from this measure, there was a 100% completion of scales among those completing follow‐up.

## Discussion

The main aim of this study was to understand the feasibility and acceptability of MUSE to service‐users and therapists. Recruitment and retention rates from this study met the green zone criteria and suggested that it was feasible to recruit to the study and retain people in it. Moreover, adherence data indicated that participants worked on hallucination‐related content. From this it was evident that MUSE was acceptable to ARMS service‐users. Also, high scores on the STTS suggested good satisfaction with the treatment and therapist, which was supported by the qualitative feedback. 95% of participants said they would recommend the intervention to other service‐users and there was very positive feedback on the impact of the treatment, with several comments suggesting that MUSE had succeeded in normalizing hallucinations and creating an alternative explanation of the experiences that reduced distress and improved functioning. Therapist feedback was positive and suggested that they had found MUSE easy to use, it had improved communication and engagement, and that they felt it had improved their knowledge and the quality of their formulations.

The measures showed a consistent reduction in scores at post‐treatment, suggesting that they can demonstrate change. There was a promising signal of efficacy, with most measures showing a large effect size, although the absence of a control group means that this could be regression to the mean or improvement over time, so no claims can be made about the effectiveness of the treatment. MUSE directly targets hallucinations, but there were reductions in post‐treatment scores for depression, anxiety and functioning, suggesting that the changes in hallucinations may have generalized to other areas. The limited scope of this feasibility trial meant that we were not able to track conversion to psychosis over an appropriate follow‐up period. One participant converted to psychosis during the trial.

NICE guidance (2014) recommends that people meeting ARMS criteria should be referred for specialist assessment and offered CBT, with or without family interventions and treatment for co‐morbidities such as anxiety or depression. However, Fusar‐Poli et al. (2019) suggest that there is not sufficient evidence to recommend any specific treatment for ARMS, so there is a pressing need to develop effective treatments in this area, particularly as current NHS policy in England is to ensure that ARMS services are commissioned (NHS Long Term Plan, 2019). CBT involves developing a formulation of the individual and would often include identifying significant factors, such as childhood sexual abuse and how this may contribute to the development of psychosis (Hardy, 2017). MUSE attempts to create a more detailed understanding of the psychological mechanisms that underlie voice‐hearing, and therefore creates a more detailed formulation with additional treatment options. MUSE is therefore fully compatible with CBT, with most therapists offering further CBT based interventions after completing MUSE. The modular structure of MUSE enables skilled clinicians to flexibly use it as part of a CBT intervention or as a stand‐alone intervention. Moreover, MUSE is also suitable for use by non‐therapists and further research is planned to assess the feasibility of non‐therapists delivering MUSE, which could significantly increase access.

Interestingly, the results also supported the novel aspects of MUSE. Firstly, the adherence checklist showed that therapists used the subtyping structure to tailor their interventions, with Inner Speech being the most used module, consistent with previous research on the frequency of subtypes (Garwood, Dodgson, Bruce, & McCarthy‐Jones, 2015). The feedback from several of the participants directly stated the benefits of this approach. Secondly, both therapists and service‐users commented that MUSE made more understandable complex psychological models that may be implicated in the development of hallucinations. This knowledge can help strengthen the credibility and acceptability of the explanation offered by the therapists as to why people see or hear things (Currell, Christodoulides, Siitarinen, & Dudley, 2016). However, some participants still found the videos ‘boring’ suggesting that the presentation of information could still be improved. Thirdly, several benefits of using a tablet were outlined, including helping to make complex theories accessible, that the modular structure promoted consistency and helped in the development of compelling formulations. An unexpected benefit was that service‐users found information being presented on a tablet, rather than by a therapist, more believable.

The reformulation of hallucinations may impact on the progression of hallucinations, through reducing frequency, distress and preventing the development of delusional explanations for the experience (Maher, 1974), all factors that increase the scores on key diagnostic criteria (PANSS, Kay, Fiszbein, & Opler, 1987; CAARMS, Yung et al., 2005). Previous research had suggested that people with strong delusional beliefs, were less likely to benefit from this approach (Dodgson et al., 2020), but in this study only a small number of participants had delusional beliefs, as rated on the PSYRATS, and these scores had reduced at follow‐up, suggesting that MUSE may be particularly suited to the ARMS group. Indeed, this detailed psychoeducation may also have value earlier in the development of hallucinations and may be used to explain anomalous sensory experiences before they become entrenched.

There were several limitations to this study. The absence of a control group precluded testing randomization and blinding procedures and makes it impossible to interpret any signal of efficacy. Recording sessions and rating them for adherence would have formed a better measure of adherence than therapist’s ratings of the modules they have used. However, the modular structure of MUSE is likely to promote adherence to the treatment. Nor was the study able to collect key outcome data on conversion to psychosis and health resource utilization.

In conclusion, this study demonstrated that MUSE was acceptable to participants meeting the green criteria for recruitment and retention, and qualitative feedback was positive about the intervention. Qualitative feedback supported the underlying assumptions of the intervention, in that subtyping of hallucinations, drawing on possible mechanisms from current psychological theories, which are made accessible through using technology, can have benefits for service‐users, particularly at the early stage of experiencing unusual sensory experiences.

## Conflicts of interest

There were no conflicts of interest. The corresponding author had full access to all the data in the study and had final responsibility for the decision to submit for publication.

## Author contributions

G. Dodgson (Conceptualization; Formal analysis; Investigation; Methodology; Resources; Supervision; Writing – original draft; Writing – review & editing) C. Aynsworth (Formal analysis; Resources; Writing – review & editing) K. Mitrenga (Software; Writing – review & editing) C. Gibbs (Methodology; Software; Writing – review & editing) V. Patton (Formal analysis; Writing – review & editing) C. Fernyhough (Conceptualization; Funding acquisition; Software; Writing – review & editing) R. Dudley (Conceptualization; Methodology; Resources; Software; Writing – review & editing) C. Ewels (Investigation; Project administration; Supervision; Writing – review & editing) L. Leach (Investigation; Methodology; Project administration; Software; Writing – review & editing) B. Alderson‐Day (Conceptualization; Data curation; Formal analysis; Investigation; Methodology; Project administration; Software; Writing – review & editing) S. Common (Conceptualization; Investigation; Methodology; Project administration; Software; Writing – review & editing).

## Supporting information

**Appendix S1**. MUSE Modules and Structured Interview Topics.

## Data Availability

The data that supports the findings of this study can be found here: https://osf.io/h9rsp/?view_only=41e82d95b80e4be08f1242faa5b729f3.
