# Supplementary material for: Managing unusual sensory experiences: A feasibility trial in an At Risk Mental States for psychosis group
Source: Psychol Psychother. 2020 Dec 15;94(3):481–503. doi: 10.1111/papt.12323 (PMC8451773; doi:10.1111/papt.12323)
Supplement: Supplementary file 1 — Appendix S1. MUSE Modules and Structured Interview Topics. [file PAPT-94-481-s001.docx]

**MUSE modules and topics:**

**Module 1 -** What are voices?

**Topics**

1. What are voices?
2. How many people hear voices?
3. Why does it become a problem?
4. Can things get better?
5. Personal experiences

**Module 2** – How the mind works

**Topics**

1. Thoughts and senses
2. How thoughts work
3. Embarrassing thoughts
4. The power of attention
5. How we use expectation

**Module 3** – Assessment

**Topics**

1. Types of unusual sensory experiences
2. What kind of voices do we hear?

**Module 4 –** Inner speech

**Topics**

1. What is inner speech?
2. Our inner speech can do amazing things
3. Why do people not recognise voices?
4. Thoughts are hard to control
5. Blocking the loop
6. Inner speech – what is the evidence?
7. Tracking the self – was that me?
8. Writers and voice-hearing
9. Imaginary friends
10. Formulation
11. Voices and relationships
12. Transforming the voice
13. Testing out your explanations
14. Living well with voices

**Module 5 –** Memory-based voice

**Topics**

1. Memory, dissociation, trauma
2. The importance of trauma
3. Threat system and soothing system
4. Formulation
5. Treating trauma

**Module 6** – Hypervigilance

**Topics**

1. Nature vs. nurture
2. Filling in the gaps
3. What our perception system is designed to do
4. Response to danger
5. Formulation
6. Threat system and soothing system
7. Mistrust

**Module 7 –** Seeing visions

**Topics**

1. Is seeing believing?
2. What do your visions mean to you?
3. Perception system design
4. Filling in the gaps
5. Tracking the self – was that me?
6. Imaginary friends
7. Testing distressing appraisals
8. Changing images

**Module 8** – Sleep

**Topics**

1. Why do we sleep?

**Structured Interview Topic – Service-User participants**

Thank you for agreeing to answer some questions about your experience of the therapy. We want to understand how people found the approach and if there was anything that you would suggest we change. We want to record the interview so we can see if any themes emerge about the treatment and might also use some anonymous quotes from people in the next stage of the research. You are welcome to say as much or as little as you like to each question, or not answer at all. Are you ready for us to begin?

My first questions are about the sessions you did with the therapist involving the smart tablet….

What was the intervention like for you?

How was it using the computer tablet?

Were there any particular things about the session that were helpful for you?

Were there any particular things about the session that were unhelpful for you?

Is there anything you would change about it?

Did it help you make sense of any of the unusual experiences you have?

Do you think the tablet influenced how easy it was to form a strong working relationship with your therapist?

Would you recommend the intervention to someone else with similar experiences?

My next questions are more about taking part in the research

.

When you started the study, we asked you to tell us about some of your experiences in an interview, and to fill out some questionnaires. What was that like for you? Is there anything you think should be done differently?

In our future research, we might need to run studies where people get “randomised” at the start – so some people would get to use the tablet, and some people wouldn’t. How would you feel about this?

If you were in a trial where you might randomly get placed in a part of the research where you get treatment as usual, or the new treatment, would you have agreed to participate?

Are there any other comments you would like to make about the research?

Structured Interview Topic – Staff participants

Thank you for agreeing to answer some questions about your experience of the therapy. We want to understand how people found the approach and if there was anything that you would suggest we change. We want to record the interview so we can see if any themes emerge about the treatment and might also use some anonymous quotes from people in the next stage of the research. You are welcome to say as much or as little as you like to each question, or not answer at all. Are you ready for us to begin?

My first questions are about the sessions you did with the service user involving the smart tablet….].

What was the intervention like for you?

How was it using the computer tablet?

Were the tablets easy to use in the sessions?

Were there any particular things about the session that were helpful for you?

Were there any particular things about the session that were unhelpful for you?

Is there anything you would change about it?

Did it help you make it easier to build a formulation of the service users unusual experiences?

Do you think the tablet influenced how easy it was to form a strong working relationship with your therapist?

Would you recommend the intervention to another therapist?

My next questions are more about taking part in the research

.

Did the research procedure run smoothly? Would you suggest making any changes to it?

In our future research, we might need to run studies where people get “randomised” at the start – so some people would get to use the tablet, and some people wouldn’t. How would you feel about this?

If you participated in the next stage of the research and were asked to be a treatment as usual therapist, do you think you would be able to stop yourself using the ideas from the treatment?

Are there any other comments you would like to make about the research?
